# Suppression of astrocytic autophagy by αB-crystallin contributes to α-synuclein inclusion formation

**DOI:** 10.1186/s40035-018-0143-7

**Published:** 2019-01-18

**Authors:** Shen-zhao Lu, Yong-shun Guo, Pei-zhou Liang, Shu-zhen Zhang, Shu Yin, Yan-qing Yin, Xiao-min Wang, Fei Ding, Xiao-song Gu, Jia-wei Zhou

**Affiliations:** 1 Institute of Neuroscience, State Key Laboratory of Neuroscience, CAS Center for Excellence in Brain Science and Intelligence Technology, Shanghai Institutes for Biological Sciences, Chinese Academy of Sciences, Shanghai, 200031 China; 2 School of Future Techology, University of Chinese Academy of Sciences, Beijing, 100049 China; 3 Center for Brain Disorders Research, Capital Medical University and Center of Parkinson’s Disease, Beijing Institute for Brain Disorders, Beijing, 100053 China; 4 Co-innovation Center of Neuroregeneration, School of Medicine, Nantong University, Nantong, 226001 Jiangsu China

**Keywords:** αB-crystallin, Astrocytes, Parkinson’s disease, Autophagy

## Abstract

**Background:**

Parkinson’s disease (PD) is characterized by a chronic loss of dopaminergic neurons and the presence of proteinaceous inclusions (Lewy bodies) within some remaining neurons in the substantia nigra. Recently, astroglial inclusion body has also been found in some neurodegenerative diseases including PD. However, the underlying molecular mechanisms of how astroglial protein aggregation forms remain largely unknown. Here, we investigated the contribution of αB-crystallin (CRYAB), a small heat shock protein, in α-synuclein inclusion formation in astrocytes.

**Methods:**

Small interfering RNA (siRNA)-mediated CRYAB (siCRYAB) knockdown or CRYAB overexpression was performed to investigate the impact of CRYAB on the autophagy in human glioblastoma cell line U251 cells. Co-immunoprecipitation (co-IP) and immunoblotting were used to dissect the interaction among multiple proteins. The clearance of α-synuclein in vitro was evaluated by immunocytochemistry. CRYAB transgenic mice and transgenic mice overexpressing A30P mutant form of human α-synuclein were used to examine the influence of CRYAB to α-synuclein accumulation in vivo.

**Results:**

We found that knockdown of CRYAB in U251 cells or primary cultured astrocytes resulted in a marked augmentation of autophagy activity. In contrast, exogenous CRYAB disrupted the assembly of the BAG3-HSPB8-HSC70 complex via binding with BAG3, thereby suppressing the autophagy activity. Furthermore, CRYAB-regulated autophagy has relevance to PD pathogenesis. Knockdown of CRYAB remarkably promoted cytoplasmic clearance of α-synuclein preformed fibrils (PFFs). Conversely, selective overexpression of CRYAB in astrocytes markedly suppressed autophagy leading to the accumulation of α-synuclein aggregates in the brain of transgenic mice expressing human α-synuclein A30P mutant.

**Conclusions:**

This study reveals a novel function for CRYAB as a natural inhibitor of astrocytic autophagy and shows that knockdown of CYRAB may provide a therapeutic target against proteinopathies such as synucleinopathies.

## Background

Parkinson’s disease (PD) is the most common movement disorder. It is characterized by chronic degeneration of nigral dopaminergic (DA) neurons. There is currently no cure for this devastating neurodegenerative disorder. This is largely due to a limited understanding of its pathogenesis. Over the decades, several hypotheses about PD pathogenesis have been proposed, including mitochondria dysfunction, oxidative stress, abnormal protein aggregation, protein kinase dysfunction, unbalanced calcium homeostasis and neuroinflammation [1]. Abnormal protein aggregation in the remaining nigral DA neurons, known as a Lewy body, is one of the most prominent pathological features in the Parkinsonian brain, in addition to the chronic loss of DA neurons. Over the past few decades, numerous studies have mainly been focused on analyzing protein accumulation and degradation in neuronal cells. This focus is largely attributable to the fact that abnormal aggregates containing disease-causing protein in neuronal cells are the most prominent pathological changes when visualizing the protein aggregates using α-synuclein antibody.

However, accumulating evidence suggests the presence of glial inclusion in PD brain. For example, Braak et al. found the presence of α-synuclein-immunoreactive inclusions in astrocytes in prosencephalic regions [2]. It is hypothesized that astrocytic protein aggregates, such as α-synuclein, are taken up from neurons to be degraded in the lysosome [3, 4]. At advanced disease stages, protein aggregates are accumulated in astrocytes. Indeed, post-mortem brain analysis showed that α-synuclein-immunoreactive inclusions appear frequently in astrocytes as well as neurons in the PD brain [2, 5–8]. Accumulated protein aggregates are likely to affect astrocyte functions, thereby contributing to neurodegeneration. This notion is supported by several animal studies. For instance, the accumulation of α-synuclein induces astrocytic mitochondrial impairments [4]. Moreover, selective overexpression of an α-synuclein mutant in astrocytes leads to degenerative movement disorder in mice, accompanied by astrogliosis [9]. These data indicate that PD pathogenesis is much more complex than we previously anticipated and glial inclusion may impact on glial function during neurodegeneration thereby contribute to disease progression. It is important to investigate the question of how the glial inclusion is formed during neurodegeneration, a question which has been overlooked in this field for years.

Recent meta-analysis of genome-wide association studies (GWASs) revealed a set of novel risk loci involved in PD susceptibility, in addition to those that have been previously reported at genome-wide significance levels. These data point to a key role for autophagy and lysosomal biology in PD [10]. Interestingly, among the PD risk loci identified, some of the candidate genes, such as Bcl-2-associated athanogene 3 (BAG3), a member of the BAG family of proteins, and GTP cyclohydrolase I (GCH1), are known to be expressed in both astrocytes and neurons. The expression levels of these genes in astrocytes were preferentially upregulated in the Parkinsonian brain along with protein aggregation [11] or in response to other pathological stimuli [12, 13], implying that these candidate genes play a role in the glial pathology of PD.

Autophagy is considered as a major system targeting long-lived proteins and dysfunctional organelles for lysosomal degradation. Impaired autophagy activity and significantly increased protein aggregates are found in α-synucleinopathies as well as in tauopathies and amyotrophic lateral sclerosis (ALS) [14–18]. Moreover, a previous study has shown that a variety of susceptible genes related to degenerative diseases are associated with autophagy-lysosome regulation [19, 20]. Indeed, BAG3 and HSPB8 whose expression was upregulated specifically in astrocytes in the PD brain [11], have been shown to be able to promote autophagy, indicating that alterd autophagy process in astrocytes is involved in the failure of glial inclusion clearance in PD and may thus contribute to disease onset/progression in neurodegeneration. The persistent existence of glial inclusions in the PD brain raises a question as to whether a yet-unidentified mechanism plays a role in this process, despite the upregulation of BAG3/HSPB8-induced autophagy.

It has been shown that αB-crystallin (CRYAB), a small heat shock protein (sHSP), is mainly expressed in astrocytes and oligodendrocytes in the central nervous system [21]. CRYAB is implicated in various protein aggregation-related neurodegenerative diseases such as Alzheimer’s disease (AD), PD, ALS, tauopathies, Alexander’s disease, and prion disorders [2, 21–28]. For example, CRYAB and tau are often co-localized in glia with tau pathology [22]. The relationship between increased glial CRYAB expression and abnormal protein accumulation remains unclear. Previously, we found that expression of CRYAB was steadily increased in the ventral midbrain and striatum of an adult mouse brain with aging [29]. Moreover, we demonstrated that the presence of abnormal CRYAB-immunoreactive inclusions in astrocyte-like glial cells in the substantia nigra (SN) of the PD brain is a remarkable pathological feature of the gliosis in PD [21]. We hypothesized therefore that CRYAB may play a role in glial inclusion formation during brain aging and contribute to PD pathogenesis.

In the present study, we tested this hypothesis by investigating the effect of CRYAB on astrocytic autophagy flux in vitro and on the degradation of α-synuclein mutant protein in vivo. We showed that CRYAB functions as a potent inhibitor of astrocytic autophagy induction through interacting with BAG3, which is a key mediator for macroautophagy (hereafter referred to autophagy) induction. By inhibiting the selective process of autophagy, elevated levels of CRYAB leads to the accumulation of aggregated proteins in astrocytes that exacerbates the pathological process of neurodegenerative diseases such as PD.

## Methods

### Animals

Adult or neonatal C57BL/6 mice were from Shanghai Laboratory Animal Centre, Chinese Academy of Sciences. CRYAB transgenic mice (FVB-Tg(GFAP-CRYAB)141.6Mes/J) and SNCA^A30P^ (B6.Cg-Tg(THY1-SNCA*A30P)TS2Sud/J) transgenic mice were purchased from the Jackson Laboratory (USA). CRYAB ^Tg^ mice in a C57BL/6 (inbred) genetic background generated by 10 backcrosses were used in the entire study. They were maintained on a 12 h light/dark cycle at 23 °C with food and water available ad libitum. All procedures performed were approved by the Institutional Animal Care and Use Committee and were in accordance with the US National Institutes of Health Guide for the Care and Use of Laboratory Animals.

### Cell culture and transfection

U251 human glioblastoma cell line (Cell Bank, Chinese Academy of Sciences) were maintained in DMEM with 10% fetal bovine serum (FBS) and 100 mM glutamine at 37 °C under 5% CO_2_. Transient transfection was performed using Lipofectamine 3000 (Invitrogen, L3000–008) according to the manufacturer’s instructions. Knocking down CRYAB was performed as described previously [30]. siRNA duplexes were used (GenePharma, Shanghai, China) and their sequences were as follows: sense: r (GGC CCA AAU UAU CAA GCU A) dTdT; antisense: r (UAG CUU GAU AAU UUG GGC C) dTdG; non-silencing control siRNA: sense: r (UUC UCC GAA CGU GUC ACG U) dTdT; and antisense: r (ACG UGA CAC GUU CGGAGAA) dTdT. At 72 h after transfection, cells were harvested for further analysis.

### Plasmids, reagents, and antibodies

The cDNA of BAG3 and CRYAB were amplified using RNA extracted from HEK293 cell, followed by RT-PCR and subcloned into pLenti vector (OBIO, H139).

H_2_O_2_ (SinoPharm Chemical Reagent Co., Ltd., China. Cat. #7722-84-1). Rapamycin (Sangon Biotech, China. Cat. #A606203) and chloroquine (Sangon Biotech. Cat. #A506569) were used. The following antibodies were used: anti-LC3 (MBL, Cat. #PM036), anti-β-actin (Sigma, Cat. #A5441), anti-RPS6KB/p70S6 (Cell Signaling, Cat. #2708), anti-phospho-RPS6KB/p70S6 (Cell Signaling, Cat. #9206), anti-mTOR (Cell Signaling, Cat. #2972), anti-phospho-mTOR (Sigma, Cat. #SAB4504043), anti-SQSTM1/p62 (MBL, Cat. #PM045), anti-GAPDH (SAB, Cat. #1336), anti-CRYAB (Santa Cruz Biotech, Cat. #sc-137,143), anti-HSPB8 (Abcam, Cat. #ab4149), anti-HSC70 (Santa Cruz Biotech, Cat. #sc-7298), anti-BAG3 (Abcam, Cat. #ab47124), anti-ubiquitin (DAKO, Cat. #z0458), anti-α-synuclein (Cell Signaling, Cat. #2628), anti-GFP (Santa Cruz Biotech, Cat. #sc-9996).

### Western blot analysis and quantification

Western blot was performed as described previously [31]. Briefly, the proteins from lysed cells were denatured and loaded on sodium dodecyl sulfate polyacrylamide gels. Afterwards, the proteins were transferred to PVDF membranes, blocked in TBST (150 mM NaCl, 10 mM Tris-HCl, pH 7.5 and 0.1% Tween 20) containing 5% (*w*/*v*) skim milk, and incubated with the corresponding primary and secondary antibodies.

Peroxidase activity was detected with Omni-ECL™ FemtoLight Chemiluminescence Kit (EpiZyme, Cat. #SQ201) and visualized and digitized with ImageQuant (LAS-4000, FujiFilm, Japan). Optical densities of bands were analyzed by using Image J. Protein levels, quantified by computer analysis as the ratio between each immunoreactive band and the levels of β-actin were expressed as a percentage of vehicle-treated control.

### Co-immunoprecipitation analysis

To analyze protein interactions in vitro, approximately 5 × 10^6^ U251 cells were harvested after 48 h transfection and lysed in 0.5 ml of RIPA buffer (50 mM Tris (pH 7.4), 150 mM NaCl, 1% NP-40, 0.5% sodium deoxycholate, 0.1% SDS). Pre-cleared lysates were subjected to immunoprecipitation with indicated antibodies directed against the epitope tag as described previously. Precipitated proteins were eluted from the beads by boiling in SDS sample buffer, and separated by SDS-PAGE. Immunoblot assays were performed using the indicated antibodies. The following primary antibodies were used: anti-Flag Affinity gel (Genomics Technology, Cat. #SG4510–10), anti-c-Myc AC (Santa Cruz Biotech, Cat. #sc-40 AC).

### Primary astrocytic culture and plasmid transfection

Astrocytes were prepared from the cerebrocortex of wild-type C57BL/6 mice at P0, as described previously [32]. The neonatal cortex was trypsinized and dissociated. Cells were then plated at 75 cm^2^ flask (Corning, USA) in DMEM containing 10% FBS. Culture media were changed 24 h later and subsequently twice a week. Cultured cells were shaken to remove the top layer of cells sitting over the astroglial monolayer to yield mainly type I astrocytes with a flat morphology between day 5 and 7 in vitro. Before experimental treatments, astrocytic cultures were passaged once. Cells were allowed to reach 90% confluence. Cultures were transfected using lentivirus, which can express CRYAB. The cells transfected with the control lentivirus were included as controls in all experiments.

### Immunostaining and confocal microscopy

For immunostaining, cells were fixed in 4% paraformaldehyde followed by 0.1% Triton-X100 in PBS (pH 7.4). After washing twice with PBS, they were incubated in FBS (PBS, pH 7.4, containing 5% FBS) to block nonspecific sites of antibody adsorption. Then, the cells were incubated with corresponding primary antibody followed by incubation of secondary antibody conjugated with either Alex488 or Alex555. The same sample were then incubated with another corresponding primary and secondary antibodies. Each sample was incubated with Hoechst to show the nucleus. The cells were imaged with invert confocal microscope (FV1000; Olympus).

For quantification of the number of autophagosomes (diameters 0.3–1.0 μm), a total of 50 cells were analyzed using ImageReader software (Fujifilm). Quantification of LC3 and BAG3 fluorescence intensity and analysis of protein co-localization coefficients in 30 cells were performed using ImageReader software (FujiFilm).

### Electron microscopy

Electron microscopic analysis of U251 cells was performed using a standard protocol. In brief, cells were fixed in 2.5% glutaraldehyde in 0.1 M PB (pH 7.4), and post-fixed in aqueous 1% OsO_4_ followed by 3–4% uranyl acetate. After ethanol and acetone dehydration and embedding in resin, ultrathin (70 nm) sections were then stained with 2% uranyl acetate followed by 0.3% lead citrate. Sections were imaged using transmission electron microscope (Nippon Tekno Co Ltd., model JEOL-1230) at 80 kV. For autophagic vacuole quantification, 20 cells, primary magnification × 20,000, were taken with systematic random sampling from each sample. The cytoplasmic volume fraction of autophagic vacuoles was estimated using ImageReader software (FujiFilm).

### Extraction of RIPA-soluble and RIPA-insoluble protein fractions of mouse brain

Extraction of RIPA-soluble and RIPA-insoluble protein fractions from mouse brain tissues was described previously [33–35]. Previous studies indicated that separation of insoluble fraction was performed on older mice at least 6 months of age [32, 36]. The tissues from one-year-old CRYAB^Tg^; SNCA^A30P Tg^ mice were isolated and stored at -80 °C prior to use. Frozen brain tissues were thawed on ice and homogenized in 10 mL/g (*v*/*w*) radio-immunoprecipitation assay (RIPA) buffer (150 mM NaCl, 1% TritonX-100, 0.1% SDS, 0.5% sodium deoxycholate, 50 mM NaF, 1 mM EDTA, 50 mM Tris, pH 7.5 with phosphatase and protease inhibitor cocktails). The homogenates were centrifuged at 14,000 g at 4 °C for 30 min and the supernatants were collected as the RIPA-soluble fraction. The pellet was washed with RIPA buffer and centrifuged at 14,000 g at 4 °C for 10 min for three times. The resultant pellet was sonicated in 5 mL/g (v/w) urea buffer (8 M urea, 100 mM NaCl, 1 mM EDTA, 50 mM Tris, pH 8.0) as urea-soluble fraction.

### Alpha-synuclein PFFs exposure

Exposure of U251 cells to α-synuclein PFFs was performed as described previously [4, 37]. Briefly, U251 cells were exposed to 0.5 μM α-synuclein PFFs (a gift from Drs. Lee, Trojanowski and Luk, USA) for 24 h. After the treatment, cells were washed twice with culture medium to remove α-synuclein PFFs. On day 0 and 3 in vitro after exposure, cells were fixed for further analyses.

### Statistical analysis

Statistical analysis was performed using GraphPad software (GraphPad Prism v6.0; GraphPad Software). All the statistical data were presented as mean ± SEM. Statistical significance of the differences was determined using Student *t*-test. Differences were considered significant at values of *P* < 0.05.

## Results

### CRYAB inhibits astrocytic autophagy

**Fig. 1:**
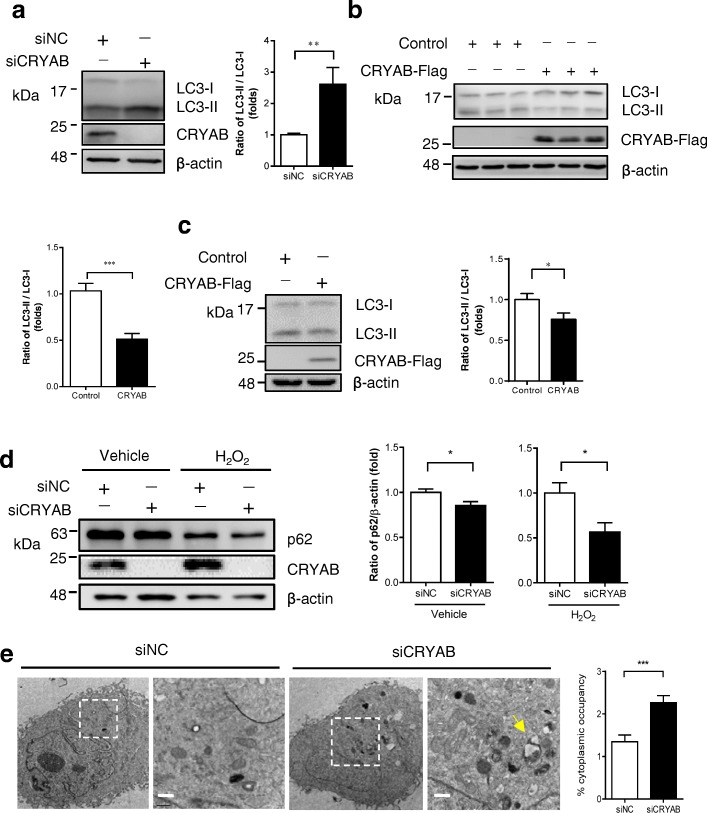
CRYAB regulates autophagy process in vitro. **a** Knockdown of CRYAB enhances the conversion of LC3-I to LC3-II in U251 cells. U251 cells were transfected with CRYAB siRNA (siNC: the siRNA of negative control; siCRYAB: the siRNA of CRYAB) and the cellular LC3 levels were assessed by Western blotting 48 h after transfection. Data are presented as mean ± SEM, *n* = 6. Unpaired *t*-test. **P < 0.01. **b** Overexpression of CRYAB inhibits LC3-II formation in U251 cells. The cellular LC3 levels were assessed by western blotting 48 h after transfection. Data are presented as the mean ± SEM, *n* = 3. Unpaired *t*-test. **P < 0.01. **c** CRYAB overexpression in primary astrocytes inhibits LC3-II formation. Primary cultured astrocytes were transfected with lentivirus vector encoding CRYAB. The cellular LC3 levels were assessed by Western blotting 48 h after transfection. Data are presented as mean ± SEM, *n* = 3. Paired *t*-test. *P < 0.05. **d** U251 cells were transfected with siCRYAB in the presence or absence of H_2_O_2_ (1 mM, 6 h). The cellular p62 levels were assessed 48 h after transfection using western blotting. Data are presented as mean ± SEM, *n* = 3. *P < 0.05. **e** Knockdown of CRYAB leads to a marked increase in the number of autophagic vacuoles. Autophagic vacuoles in U251 cells transfected with siNC or siCRYAB were examined using electron microscopy. The arrows indicate autophagic vacuoles. Scale bars: 0.5 μm. The graph shows a statistical analysis of cytoplasmic occupancy of autophagic vacuoles in the cells. Data are presented as mean ± SEM. Twenty cells in each group were examined. Unpaired *t*-test. ***P < 0.001

To investigate whether CRYAB has an impact on autophagy in astrocytes, we performed small interfering RNA (siRNA)-mediated CRYAB (siCRYAB) knockdown in U251 cells, which is a cell line derived from human glioblastoma. The conversion of LC3-I to LC3-II was markedly enhanced by CRYAB knockdown (Fig. 1a). In contrast, the overexpression of CRYAB in U251 cells remarkably reduced the conversion of LC3-I to LC3-II in U251 cells compared with cells transfected with an empty vector (Fig. 1b). Consistent with these results, the reduced conversion of LC3-I to LC3-II was also observed in primary cultured astrocytes overexpressing CRYAB via lentivirus-mediated transfection compared with the control (Fig. 1c). These results suggest that CRYAB is a potent regulator of autophagy in astrocytes under normal conditions.

We then asked whether CRYAB is capable of influencing autophagy in astrocytes under stress conditions. Hydrogen peroxide (H_2_O_2_) has been widely used to induce autophagy in vitro [38]. We evaluated the autophagy activity by measuring p62 levels by using immunoblotting. p62 is known as an autophagy substrate which serves as a reporter of autophagy activity. Promotion of autophagy induced by knockdown of CRYAB resulted in a significant reduction (~ 15%) in p62 levels compared with control (Fig. 1d). Exposure to H_2_O_2_ of U251 cells in combination with CRYAB knockdown led to a further reduction in p62 levels (~ 45%), as compared to H_2_O_2_ treatment alone. These results suggest that CRYAB also inhibits autophagy in U251 cells under pathological conditions.

To validate whether CRYAB knockdown truly induces autophagy, we performed electron microscopy analysis on U251 cells. It was revealed that knockdown of CRYAB induced robust formation of autophagic vesicles, mature autophagic vesicles and autolysosomes with visible cytoplasmic contents. The total cytoplasmic occupancy of the intracellular autophagic vacuoles was doubled in siCRYAB-treated cells compared with the control (Fig. 1e). Taken together, these data indicate that CRYAB is a crucial autophagic regulator in astrocytes.

### CRYAB inhibits autophagy in a mTOR-independent manner

**Fig. 2:**
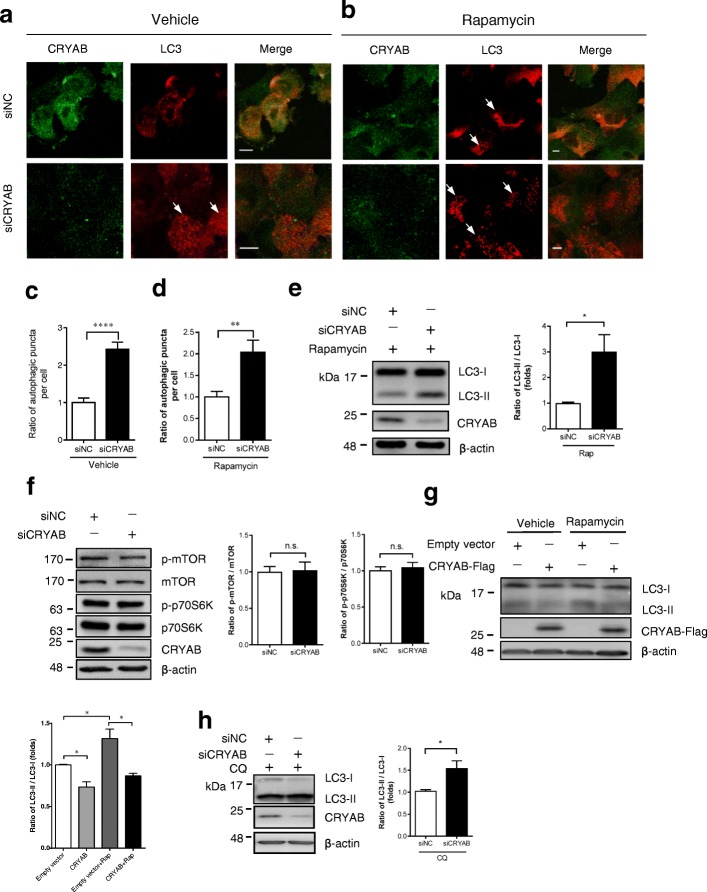
CRYAB inhibits autophagy in a mTOR-independent manner. **a**-**b** Knockdown of CRYAB in U251 cells enhances the formation of LC3^+^ dots. U251 cells were transfected with siCRYAB. Some of these cells were also treated with 1 μM rapamycin for 6 h. The cells were immunostained with CRYAB and LC3 antibodies 48 h after transfection. Scale bars: 5 μm (**a**) and 10 μm (**b**). **c**-**d** Quantitative data in a and b, respectively. Data are presented as the mean ± SEM, c, *n* = 50 cells, d, *n* = 40 cells. Unpaired t-test. *P < 0.05. **e** Treatment with rapamycin does not alter CRYAB knockdown of CRYAB-induced augmentation in the conversion of LC3-I to LC3-II in U251 cells. U251 were transfected with siCRYAB and stimulated with 1 μM rapamycin for 6 h. At 48 h after transfection, the cellular LC3 levels were assessed by western blot. The graph shows a statistical analysis of the conversion of LC3-I to LC3-II in the cells. Data are presented as mean ± SEM, *n* = 5. **f** Immunoblot analysis of the cell lysates from U251 cells transfected with siCRYAB. The graph shows a statistical analysis of the expression levels of phosphorylated mTOR, p70S6K and total mTOR. Data are presented as mean ± SEM, *n* = 3. *P < 0.05. **g** Immunoblot analysis of the cell lysates from U251 cells overexpressing CRYAB and treated with rapamycin (1 μM, 6 h). Data are presented as mean ± SEM, *n* = 3. *P < 0.05. **h** Knockdown of CRYAB-induced enhancement in autophagy does not affect autophagic degradation and autophagosome-lysosome fusion. Western blotting analysis of the conversion of LC3-I to LC3-II in U251 cells transfected with siCRYAB and stimulated with 10 μM CQ for 6 h. Data are presented as mean ± SEM. *n* = 3. *P < 0.05

Next, we sought to determine the mechanisms by which CRYAB regulates autophagy. It is well known that mTOR is an important modulator of autophagy [39]. To investigate whether the CRYAB-mediated inhibition of autophagy is dependent on the mTOR pathway in astrocytes, we assessed the effect of rapamycin, an inhibitor of mTOR on autophagy, in U251 cells treated with and without siCRYAB. As expected, siCRYAB strongly elevated autophagy levels, as evidenced by significant increases in the number of LC3-positive puncta (Fig. 2a, c) compared with that number in untreated cell. Concomitant treatment with rapamycin further elevated autophagy levels, as shown by the number of LC3-positive puncta (Fig. 2b, d) and the ratio of LC3-II / LC3-I (Fig. 2e). In contrast, CRYAB knockdown did not profoundly alter the levels of phosphorylated mTOR and phosphorylated p70S6K (Fig. 2f). These data indicate that the molecular mechanisms responsible for CRYAB knockdown-mediated promotion of autophagy is distinct from a well-established mechanism found in rapamycin-treated cells. Interestingly, CRYAB overexpression profoundly attenuated rapamycin-induced autophagy (Fig. 2g).

Moreover, we determined whether CRYAB suppresses autophagy through the inhibition of autophagosome-lysosome fusion. We found that in the presence of chloroquine (CQ), an inhibitor for the autophagosome-lysosome fusion, CRYAB knockdown further augmented the accumulation of autophagosomes compared with CQ treatment alone (Fig. 2h), suggesting that CRYAB does not regulate autophagy through the inhibition of autophagolysosome degradation. Taken together, these results suggest that CRYAB may regulate autophagy in the upstream of mTOR pathway.

### CRYAB inhibits BAG3-dependent autophagy via disruption of the assembly of chaperon complex BAG3-HSPB8-HSC70

**Fig. 3:**
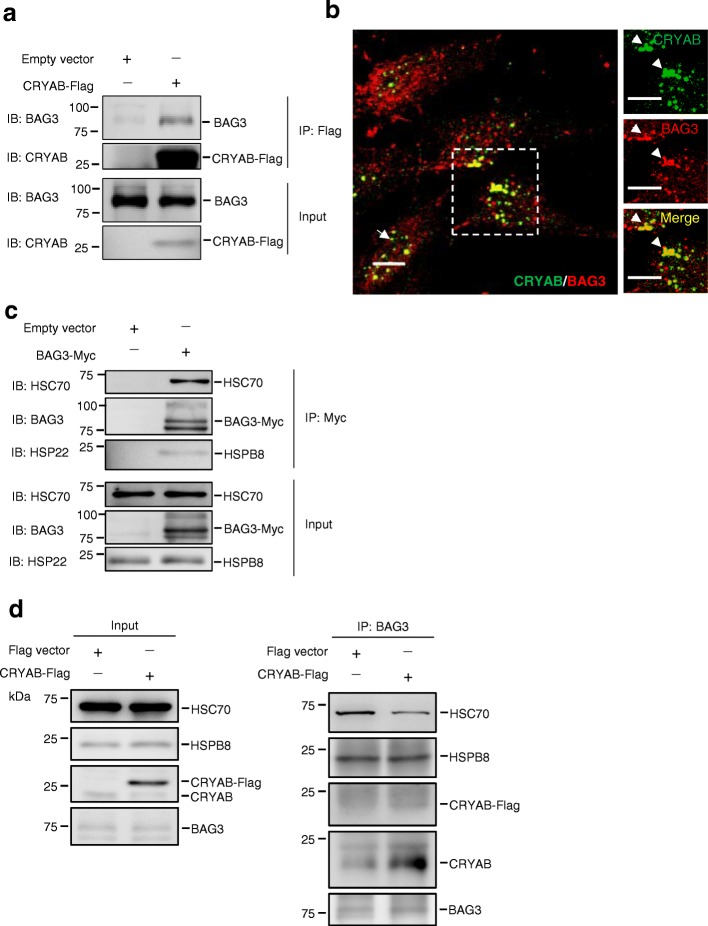
Overexpression of CRYAB inhibits binding between BAG3 and HSC70 in vitro. **a** Co-immunoprecipitation (co-IP) reveals that CRYAB interacts with BAG3 in U251 cells. Data are from three independent experiments. **b** Double immunofluorescence staining. Some CRYAB^+^ (green) cells (arrowheads indicate examples) express BAG3 (red) in the U251 cell cultures. Right panel is an enlarged view of the box in the left panel. Scale bars: 10 μm. **c** Co-IP analysis reveals that BAG3 binds to HSC70 and HSPB8. **d** Co-IP analysis reveals that CRYAB overexpression inhibits binding between BAG3 and HSC70 in U251 cells. Data are from three independent experiments

Recent studies have shown that BAG3 potently stimulates macroautophagy [40–43], an effect that resembles the phenotypes observed in CRYAB knockdown cells (Fig. 1a). Moreover, BAG3 is known to bind to wild-type CRYAB and CRYAB mutant R120G to inhibit CRYAB mutant-induced toxicity by degrading the mutant CRYAB in rat neonatal cardiomyocytes in an autophagy-independent manner in this setting [44]. These findings led us to hypothesize that CRYAB may be functionally related to the BAG3 signalling of autophagy regulation in astrocytes. To test the hypothesis, we first verified the association between BAG3 and CRYAB in astrocytes. As predicted, co-immunoprecipitation (co-IP) assays using cell lysates from U251 cells stably overexpressing CRYAB-Flag revealed that the two proteins bound to each other (Fig. 3a). Immunocytochemical staining showed the co-localization of CRYAB and BAG3 in cultured U251 cells (Fig. 3b).

Moreover, previous studies have shown that BAG3-mediated autophagy is required by its interaction with HSC70 and HSPB8 [42, 43]. It was observed that BAG3 did bind to HSC70 and HSPB8 in U251 cells (Fig. 3c). Thus, we speculated that CRYAB may bind to BAG3 and competitively inhibit the formation of BAG3-HSPB8-HSC70 complex. To test this, we performed BAG3 co-IP assays using U251 cell lines stably overexpressing CRYAB-Flag or empty vector as control. It was found that HSC70, HSPB8 and CRYAB could be detected in BAG3 immunoprecipitates and the levels of HSC70 were decreased in the immunoprecipitates from cells overexpressing CRYAB (Fig. 3d-e). These results suggest that elevation of CRYAB levels can disrupt the assembly of the protein complex BAG3-HSPB8-HSC70.

**Fig. 4:**
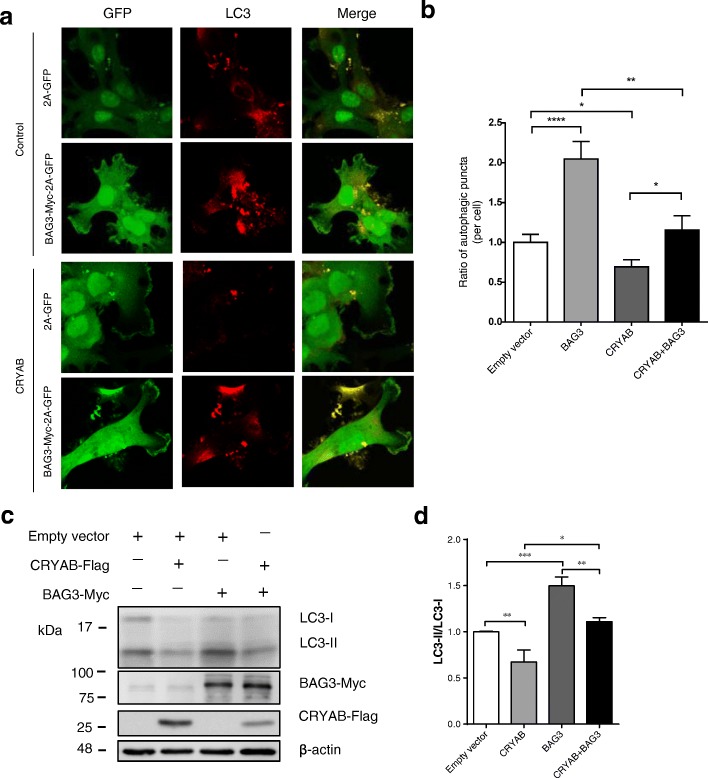
CRYAB inhibits the BAG3-dependent autophagy in U251 cells. **a** U251 cells expressing CRYAB stable were transfected with plasmids encoding BAG3-Myc-2A-GFP. The cultures were fixed 48 h after transfection and immunostained with GFP and LC3 antibodies. Scale bars: 10 μm. **b** Quantification of the number of LC3^+^ dots per cell shown in (A). Data are presented as mean ± SEM, *n* = 30 cells. **c**, **d** Representative immunoblot shows that BAG3 overexpression elevates the conversion of LC3-II to LC3-I in U251 cells. Cells were transfected with BAG3 plasmids for 48 h. Data are presented as mean ± SEM, *n* = 5. Unpaired t-test. **P < 0.01

To functionally characterize the inhibitory effect of CRYAB on BAG3-dependent autophagy, we transfected U251 cells with cDNAs encoding BAG3 and CRYAB concomitantly or separately. Consistent with previous studies, autophagy was markedly increased in BAG3-overexpressing cells compared with the control (Fig. 4a-d). In contrast, BAG3-elevated autophagy was remarkably suppressed by CRYAB overexpression compared with cells transfected with BAG3 alone (Fig. 4a-d), as demonstrated by the number of LC-3-positive puncta (Fig. 4a-b) and the ratio of LC3-II / LC3-I (Fig. 4c-d). These results suggest that CRYAB is a potent inhibitor of BAG3-induced autophagy. Taken together, CRYAB binds to BAG3, preventing the formation of the BAG3-HSPB8-HSC70 complex, thereby inhibiting autophagy in U251 cells.

### CRYAB inhibits the degradation of aggregated proteins in astrocytes by regulating autophagy

**Fig. 5:**
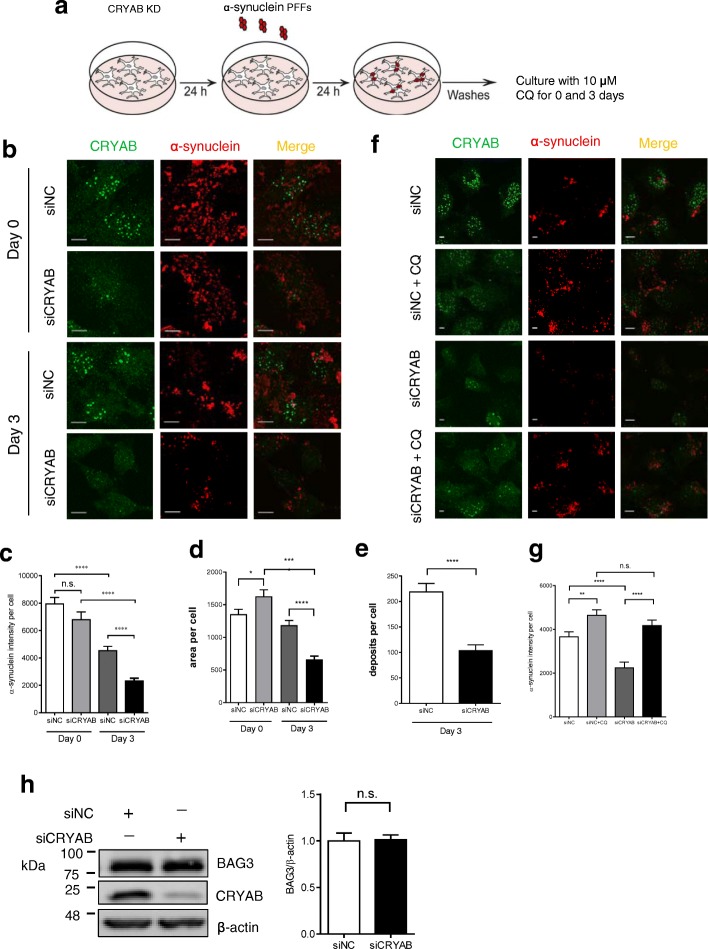
Knockdown of CRYAB in astrocytes promotes α-synuclein degradation. **a** A schematic shows the timeline of treatment of U251 cells. Cells were transfected with siRNA of CRYAB were exposed to 0.4 μM α-synuclein PFFs for 24 h, washed thoroughly, and cultured for an additional 0 and 3 days in α-synuclein-free medium with 10 μM CQ stimulation before fixation. **b**-**c** The intracellular location of the α-synuclein after ingestion in day 0 and day 3 without CQ stimulation was confirmed with confocal imaging. Scale bars: 1 μm. The graph shows a statistical analysis of the mean intensity of the intracellular α-synuclein per field in cells. Data are presented as mean ± SEM, *n* = 40. **d** The intracellular location of the α-synuclein after ingestion in day 0 and day 3 without CQ stimulation was confirmed with confocal imaging. The graph shows a statistical analysis of the mean intensity of the total area of the α-synuclein deposits per cell. Data are presented as mean ± SEM, *n* = 40. **e** The graph shows a statistical analysis of the mean intensity of the number of α-synuclein deposits per cell. Data are presented as mean ± SEM, *n* = 40. **f**, **g** The intracellular location of the α-synuclein after ingestion on day 3 with CQ stimulation was confirmed with confocal imaging. Scale bars: 1 μm. The graph shows a statistical analysis of the mean intensity of the intracellular α-synuclein per field in cells. Data are presented as mean ± SEM, *n* = 40. **h** Western blotting analysis shows that knockdown CRYAB does not affect BAG3 expression levels. Data are expressed as mean ± SEM, *n* = 3. n.s. no significance. *P > 0.05

Given that CRYAB is able to regulate autophagy in vitro as shown in the present study, we then investigated whether CRYAB has an impact on the autophagic degradation of α-synuclein in astrocytes. U251 cells were transfected with CRYAB siRNA followed by exposure to 0.4 μM α-synuclein pre-formed fibrils (PFFs) [45] for 24 h. Some cells were maintained for an additional 3 days in the absence of α-synuclein PFFs before fixation (Fig. 5a). Quantifications of α-synuclein immunosignals revealed that CRYAB knockdown accelerated the degradation of α-synuclein on day 3 following incubation with α-synuclein PFFs compared with the control, while no significant alteration of α-synuclein immunosignals was observed on day 0 in siCRYAB-treated cell cultures compared with control (Fig. 5a-e). Moreover, inhibition of autophagosome-lysosome fusion with CQ markedly reduced autophagic degradation of α-synuclein PFFs. This inhibitory effect was not significantly attenuated by CRYAB knockdown (Fig. 5a, f, g). These data suggest that CRYAB is required for the accumulation of α-synuclein in astrocytes through the inhibition of autophagy.

To determine whether the impact of CRYAB knockdown on α-synuclein degradation is attributable to enhanced expression of BAG3 which is known to promote autophagy, we assessed the expression levels of BAG3 after CRYAB knockdown in U251 cells. We found that the expression levels of BAG3 were not significantly altered after CRYAB knockdown (Fig. 5h), suggesting that CRYAB knockdown-induced augmentation of α-synuclein aggregate clearance result from elevated BAG3 activity but not its expression.

### Astrocytic CRYAB promotes the accumulation of α-synuclein in vivo

**Fig. 6:**
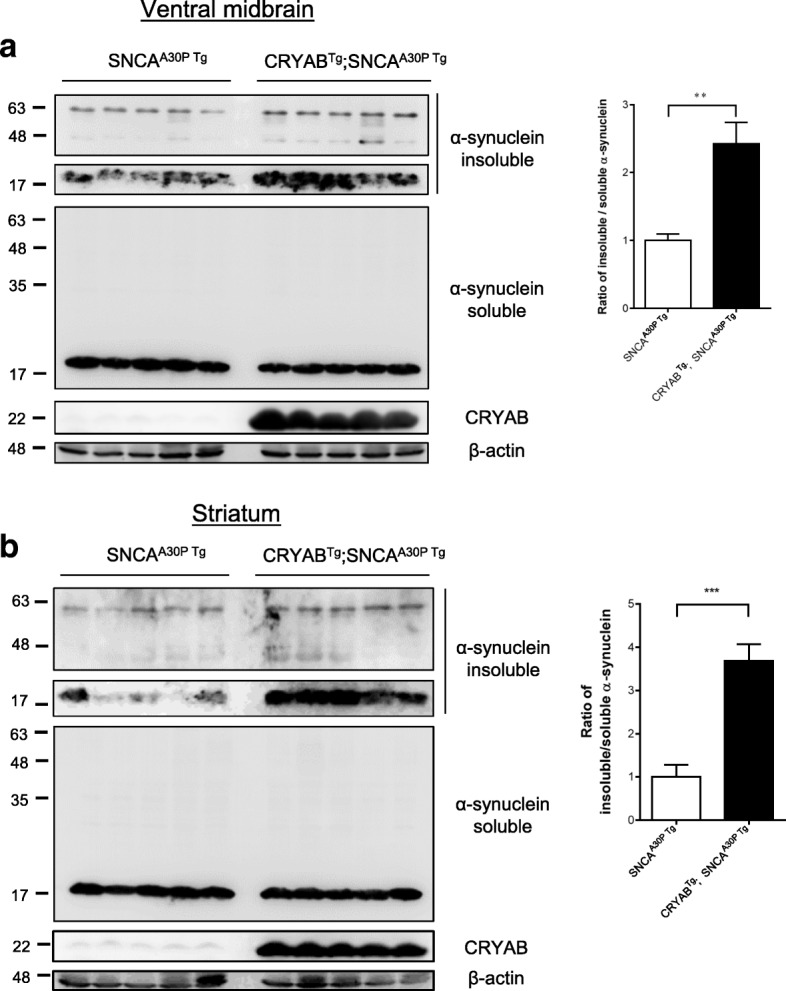
Preferential overexpression of CRYAB in astrocytes promotes protein aggregation of a-synuclein in SNCA^A30P^ mutant mouse brain. **a** Expression levels of RIPA-soluble and RIPA-insoluble protein fractions extracted from the ventral midbrain (VM) in CRYAB^Tg^ / SNCA^A30P Tg+^. The graph shows a statistical analysis of the conversion of insoluble to soluble α-synuclein in the tissue. Data are presented as mean ± SEM, *n* = 5. **b** Expression levels of RIPA-soluble and RIPA-insoluble protein fractions extracted from the striatum in CRYAB^Tg^ / SNCA^A30P Tg+^. The graph shows a statistical analysis of the conversion of insoluble to soluble α-synuclein in the tissue. Data are presented as mean ± SEM, *n* = 5

Next, we extended our investigation in vivo to evaluate the impact of CRYAB in astrocytes on the accumulation of α-synuclein using a transgenic mouse model expressing human α-synuclein mutant. We crossed transgenic mice overexpressing the hamster *Cryab* gene under the control of the human glial fibrillary acidic protein (hGFAP) promoter to target expression preferentially to astrocytes (hereafter referred to as CRYAB^Tg^) [46] with transgenic mice overexpressing A30P mutant form of human α-synuclein (SNCA^A30P Tg^) [47]. Overexpression of CRYAB markedly increased the accumulation of RIPA-insoluble α-synuclein in multiple brain regions, including the ventral midbrain, and striatum of 12-month-old double transgenic mice compared with SNCA^A30P Tg^ mice which served as control (Fig. 6a-b). These results indicate that CRYAB is sufficient for the inhibition of the autophagic degradation of α-synuclein in astrocytes.

## Discussion

**Fig. 7:**
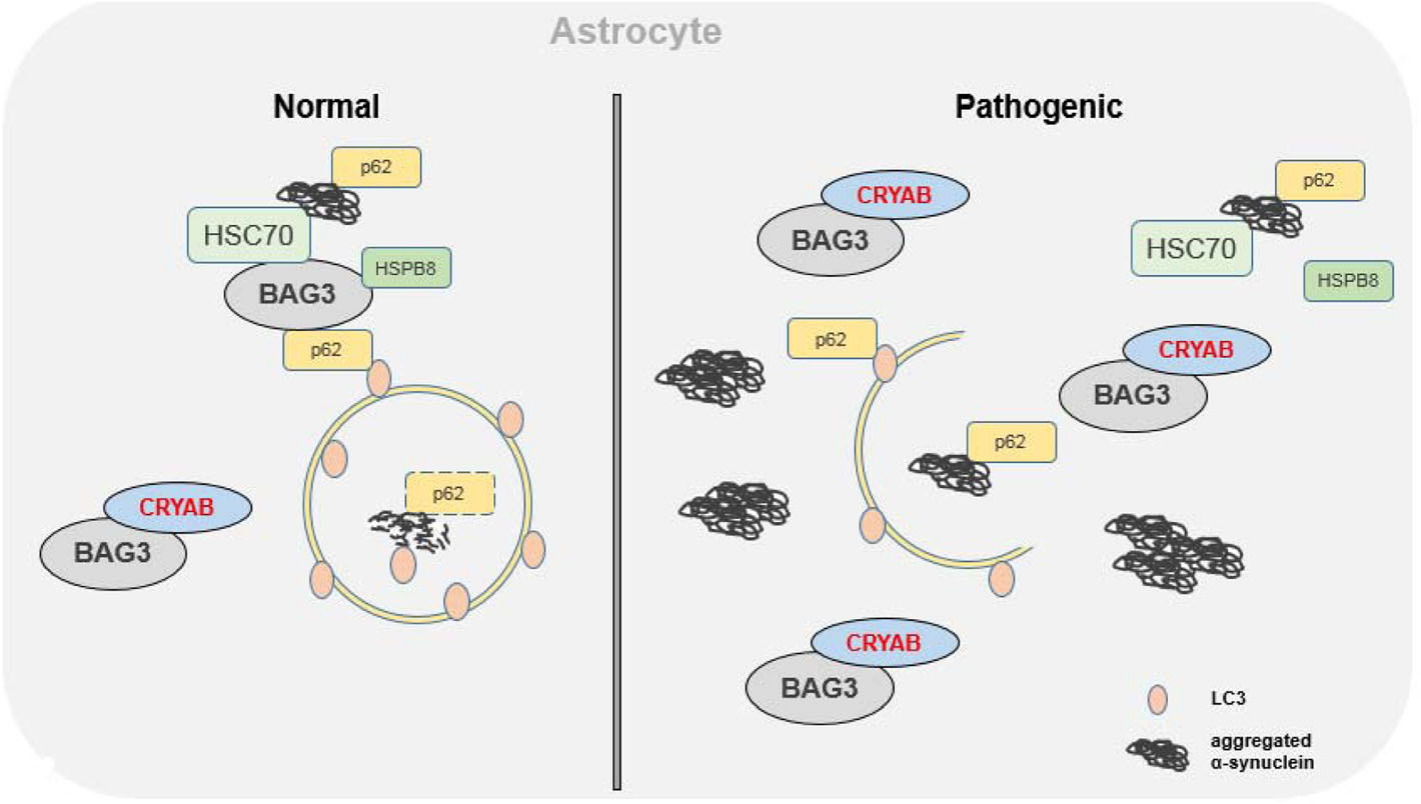
Model for the role of CRYAB in the clearance of protein aggregates in astrocytes via interaction with BAG3, HSC70 and HSPB8 under both physiological and pathological conditions. During aging and aging-related disease, deregulation of CRYAB in astrocytes may result in a suppression of autophagy leading to the accumulation of α-synuclein proteins and/or organelles

It has been well recognized that misfolded proteins and abnormal protein aggregation in neuronal cells are the hallmarks of pathology in various neurodegenerative diseases, such as AD, PD and ALS. Interestingly, accumulating evidence has suggested that abnormal protein aggregation also occurs in astrocytes, at least in the advanced stages of the diseases. However, the molecular mechanisms underlying the aberrant accumulation of disease-associated proteins remain largely unknown. In the present study, we demonstrated that CRYAB in astrocytes potently inhibits autophagy and contributes to protein aggregation and neurodegeneration. Mechanistically, CRYAB inhibited the assembly of the functional complex BAG3-HSPB8-HSC70 by binding to BAG3, leading to the deregulation of BAG3-induced autophagy (Fig. 7). These data indicate that the CRYAB-induced suppression of autophagy plays a key role in the aberrant accumulation of α-synuclein in astrocytes, which is a critical pathological event involved in the neurodegeneration of PD.

Previous studies suggest that CRYAB does not regulate autophagy in neonatal rat ventricular myocytes [48]. However, we found CRYAB to be required for the regulation of autophagy in astrocytes; as in an adult mouse brain CRYAB is enriched in astrocytes and oligodendrocytes, providing a molecular basis for the CRYAB-dependent inhibition of astrocytic autophagy. These data demonstrate the intriguing specialization of CRYAB function in the brain. CRYAB participates in the regulation of astrocytic autophagy of the brain, representing a mechanistically distinct molecular machinery utilized in the regulation of autophagy in astrocytes.

Numerous studies have shown that sHSPs, including CRYAB, are often considered to be molecular chaperones, that participate in the ubiquitin-proteasome degradation process. sHSPs help hydrolyse misfolded proteins by recognizing and combining with them, to prevent cellular toxicity under certain stress conditions [49, 50]. CRYAB also plays a role in inhibiting inflammation in the process of degenerative diseases [29, 51–53]. In the present study, we demonstrated a novel function for CRYAB in astrocytic autophagy regulation. The discrepancy between our findings in the present study and others suggests multifaceted functions of CRYAB. CRYAB plays distinct functions depending on circumstances related to the nature of insults, site of injury and disease staging. We speculate that, under normal conditions or at an early stage of PD, CRYAB functions as a molecular chaperone, participating in the ubiquitin proteasome pathway for misfolded protein degradation. Additionally, it inhibits inflammation to maintain cellular homeostasis under stress conditions. While at the advanced disease stages, despite of the increased levels of CRYAB, CRYAB is not able to prevent the progression of neuroinflammation. Meanwhile, as the protein aggregations become a more severe burden, they are released into the extracellular space and taken up by glial cells, such as astrocytes and microglia [2, 4]. Elevated levels of CRYAB in astrocytes prevent aggregated proteins from degradation by inhibiting autophagy, which in turn accelerates the degeneration of both neuronal and glial cells.

How the function of CRYAB switches is currently unclear. It may be attributable to a particular state of CRYAB determined by its phosphorylation at different sites. This is supported by recent studies showing that CRYAB in different phosphorylation forms plays distinct roles. Levels of CRYAB ser59 are increased significantly under pathological conditions and it plays a role in accelerating inflammation [54]. Therefore, it is necessary to distinguish the different phosphorylation forms of CRYAB in the various context of neurodegenerative diseases.

As shown in Fig. 6, we found that CRYAB derived from astrocytes enhanced the accumulation of insoluble α-synuclein in SNCA^A30P^ Tg mice driven by Thy1 promoter. Given that Thy1 promoter-driven expression of Cre recombinase is largely restricted to neurons [55], it raises the question as to why astrocytic CRYAB promotes α-synuclein accumulation in neuronal cells. Interestingly, a study, using luciferase reporter mice produced by Thy1-Cre mice, showed that Thy1 promoter-driven expression of luciferase reporter gene can be detected in both neurons and astrocytes [56], suggesting that Thy1 promoter is not exclusive for targeting gene expression in neurons, as previously expected. This is supported by a direct evidence showing that in SNCA^A30P^ Tg mice, accumulated α-synuclein could be detected in both astrocytes and neurons on the midbrain and brainstem sections [4]. Thus, an alternative explanation for the results shown in Fig. 6 is that astrocytic CRYAB promotes the accumulation of insoluble α-synuclein in astrocytes of SNCA^A30P^ Tg mice in a cell-autonomous fashion.

We showed in the present study that CRYAB inhibits BAG3-mediated selective autophagy which is an adaptive process to maintain protein homeostasis under stress [57, 58]. The BAG3-HSPB8-HSP70 complex promotes thedegradation of aggregated proteins which is vital for age-related neurodegenerative diseases. During aging, theexpression of BAG3 is induced under oxidative stress and plays a neuroprotective role [41]. Through inhibition of proteasome system, BAG3 can also be upregulated along with aggresome provoking [41]. Through inhibition of proteasome system, BAG3 can also be upregulated along with aggresome provoking [59]. The overexpression of BAG3 in primary cultured neurons significantly promotes Tau degradation In an ALS model, neuronal BAG3 expression is higher in the spinal cords of mutant SOD transgenic mice [43]. A misfolded proteins, such as mutant SOD can also be targeted by BAG3/HSC70, following autophagic degradation [43]. Thus, BAG3-mediated macroautophagy has been considered as a potential therapeutic target for neurodegenerative diseases. Interestingly, during aging or in degenerative diseases, BAG3-HSPB8 expression was more significantly increased in astrocytes than neurons, suggesting that astrocytic autophagy regulation is involved in neurodegeneration and actively responds to extracellular aggregates [11]. Our results showed that the selective overexpression of CRYAB in astrocytes resulted in the heavier accumulation of α-synuclein in aged mice, indicating that suppression of astrocytic autophagy can lead to the aggregation of α- synuclein. Our findings provide new insight into the role of astrocytic autophagy in the degradation of abnormal protein aggregates.

## Conclusions

In summary, our study demonstrates that CRYAB is a potent inhibitor of astrocytic autophagy via interacting with BAG3, demonstrating a new signalling pathway that negatively regulates the degradation of α-synuclein aggregates. The present study suggests that the balance between CRYAB-BAG3 and BAG3-HSPB8-HSC70 complexes in astrocytes is an integral components of neuropathology in neurodegeneration. Aberrant increases in the levels of CRYAB inhibit astrocytic autophagy, leading to more severe protein aggregation. The findings that knockdown of astrocytic CRYAB promotes the degradation of aggregated proteins suggest that CRYAB may be a new therapeutic target for future intervention in PD.

## Abbreviations

AD: Alzheimer’s disease
ALS: Amyotrophic lateral sclerosis
BAG3: Bcl-2-associated athanogene 3
co-IP: Co-immunoprecipitation
CRYAB: αB-crystallin
DA: Dopaminergic
PD: Parkinson’s disease
PFFs: Preformed fibrils
sHSPs: Small heat shock protein
siCRYAB: Small interfering RNA (siRNA) targeting CRYAB
